# Opium Usage and Risk of Head and Neck Cancer: A Systematic Review and Meta-Analysis

**DOI:** 10.31557/APJCP.2021.22.3.661

**Published:** 2021-03

**Authors:** Garima Singh, Abhishek Jaiswal, Akhil D Goel, Pankaja Raghav

**Affiliations:** 1 *All India Institute of Medical Sciences, Jodhpur, Rajasthan, India. *; 2 *All India Institute of Medical Sciences, New Delhi, Delhi, India. *

**Keywords:** Opium, head and neck cancer, oral cancer, esophageal cancer, systematic review, Afeem, Doda

## Abstract

**Introduction:**

Opium is among the most used substance of abuse worldwide. More than 50 million opium users are there worldwide, majority of whom are in Asia. Opium usage have been reported to be associated with cancer. This study aimed to find the association between opium use or abuse and head and neck cancer.

**Methods:**

A systematic search was conducted in Medline, Scopus, Cochrane, and Google Scholar database for studies published from inception till 1^st^ November 2019. Two authors independently reviewed the studies, did quality assessment, and extracted data in standardized data extraction form. Pooled estimate of OR for risk of head and neck cancer was calculated using random effects model using the method of DerSimonian and Laird, with the estimate of heterogeneity being taken from the inverse-variance model. Subgroup and sensitivity analysis were performed. The protocol was registered in PROSPERO (CRD42020156049).

**Results:**

Fourteen studies were included in data synthesis (11 case control studies and 3 cohort studies). Eleven case control studies were included in synthesizing the results for meta-analysis. Pooled odds ratio for risk of cancer among opium users for the 11-case control study was 3.85 (2.18-6.79). Heterogeneity was high (I-squared=92.0%, Tau-squared=0.88). There was no publication bias in the study. Subgroup analysis showed highest OR for pooled estimate for risk of laryngeal cancer (19.98 (11.04-36.15)).

**Conclusion:**

There was almost four-fold rise in risk of head and neck cancer among opium users compared to non-users.

## Introduction

Illegal drug use is a worldwide phenomenon, which has drastic effects on public health. Among various drugs of abuse, opiates are among the most used substances and pose a large public health problem around the world. According to world drug report 2019, approximately 53.4 million people globally had used opioids in the year 2017, which is 56 per cent more than the estimate for 2016. (World Drug Report 2019 (United Nations publication, Sales No. E.19.XI.8)., 2019).

Estimates from 2019 suggest that there are approximately 54 million opium users worldwide, 80% of whom live in Asia. Ease of access, and traditional beliefs about opium are the two factors responsible for high rate of consumption (Hosseini et al., 2010) 

Opium use or abuse leads to several acute health hazards and some reports also states association between use long term effects of opium use and risk of cancer. Link between opium and oral, bladder, lung, head and neck and gastric cancers have been found in past studies. (“American Cancer Society | Information and Resources about for Cancer,” 2019) Many experiments and case control studies have explored the carcinogenic effects of long term use of opium. (Sheikh et al., 2020)

Opium has a complex chemical composition comprising at least 25 alkaloids and other ingredients. Before consumption as opium, the harvested latex of poppy is dried and boiled. Commonly consumed forms of illicit opium include raw (or crude) opium, opium dross (tarry residues formed after smoking raw opium), and refined opium or opium sap (boiled opium dross with or without added raw opium). All three forms are either smoked or ingested. Smoking any form of opium give rise to opium pyrosylates (solid residues of combusted opium) (Warnakulasuriya et al., 2020). Various previous studies have shown carcinogenic characteristics exhibited by opium dross, and opium pyrosylates (Friesen et al., 1987, 1985; Hewer et al., 1978; Malaveille et al., 1982; Warnakulasuriya et al., 2020). 

Although most of the published studies have not assessed different types, routes and doses of opium which differ in their carcinogenicity, bioavailability and by products (Friesen et al., 1987; Shakeri et al., 2012).

CIA world factbook, states that, India is the world’s largest manufacturer of legal opium for the pharmaceutical industry. Some places where opium are grown are Pratapgarh in Rajasthan; Mandsaur, Ratlam, Neemuch in Madhya Pradesh and Barabanki, Bareily, Lucknow and Faizabad in Uttar Pradesh. (“South Asia: India-The World Factbook - Central Intelligence Agency,” 2019) Afghanistan is among the other major cultivator of opium in the world contributing almost half (Azimi, 2019). According to “Global Cancer Incidence, Mortality and Prevalence 2018 Report”, 5 years prevalence for lip and oral cavity cancer in India was 19.59%, with mortality risk of 10.09% whereas head and neck cancer in India accounted for 30% of all cancers (Bray et al., 2018).

In this systematic review and meta-analysis, we aimed to find the association between opium use or abuse and head and neck cancer. 

## Materials and Methods

Protocol and Registration: The protocol of this systematic review was registered in PROSPERO (International prospective register of systematic reviews) at www.crd.york.ac.uk under the PROSPERO-ID CRD42020156049.

The review team developed the methods for the present systematic review and meta-analysis following the PRISMA statement for preferred reporting items for systematic reviews and meta-analysis protocols (Moher et al., 2015). Box 1 depicts the research question using PECOS format. 

The present systematic review and meta-analysis has included published articles if they met all of the following criteria: original observational studies on human participants; exposure related to opium; outcome of interest is head and neck cancer; studies providing risk estimate or enough information to calculate such a measure. 

*Search strategy*

**Figure 1.**
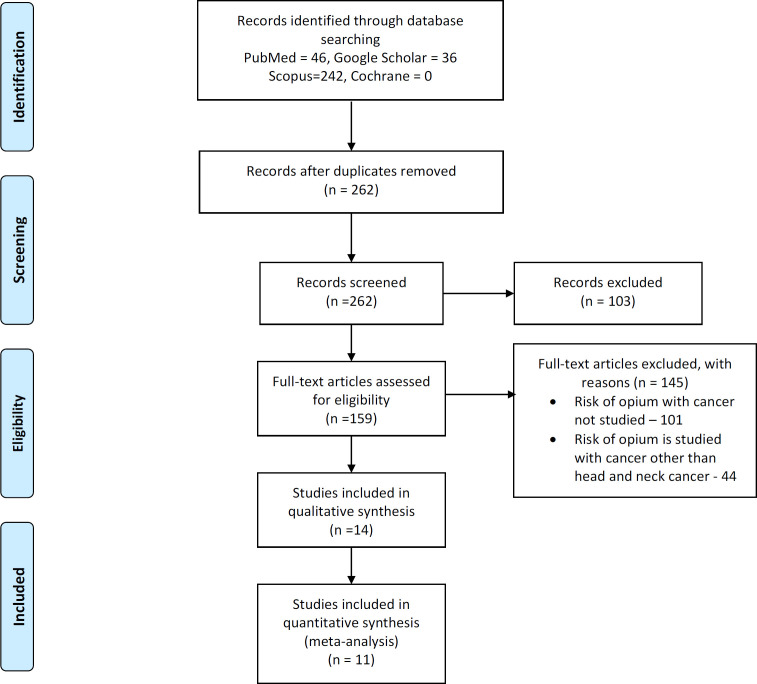
PRISMA Flowchart Showing Selection of Studies

A thorough systematic literature search was conducted in the following electronic databases namely Medline (PubMed), Cochrane library (Cochrane), Scopus, and Google scholar by two independent researchers. Articles were searched from inception till 1st November 2019. Detailed Search strategy is described in Appendix 1. Rayyan software was used for including, and excluding the articles from the records identified through search strategy. References of included articles were manually retrieved. The whole process of study selection is summarized in PRISMA in Figure 1.

*Data extraction and management*

Data was extracted and tabulated using a standardized data extraction form by two independent reviewers. Discrepancies were resolved via referencing the original article. The following data was extracted - author, year of publication, journal, country or region, study design, sample size, diagnostic criteria of head and neck cancer, number of cases and control/ exposed and non-exposed, head and neck cancer type, Opium usage, risk estimate of head and neck cancer comparing various levels of opium usage, and main outcome (Odds ratio). 

*Appraisal of quality and risk bias:*

The risk of bias was assessed based on the New Castle Ottawa Scale (Wells et al., 2014). Eleven Case control studies were analyzed for risk of bias, based on questions asked in following sections: Selection of cases and controls; Comparability of cases and controls; Ascertainment of exposure. Depending on the star rating of each questions of three sections studies were given color coding. If either of two star or both stars present then green, both absent then red and if not mentioned in the study then it is represented by yellow color. Three cohort studies were analyzed based on following questions: Selection of cohorts; Comparability of cohorts; Assessment of outcome (Wells et al., 2014). 

*Data synthesis*

The risk estimate (OR) of head and neck cancer due to opium usage was provided with 95% confidence interval (C.I.). Heterogeneity across enrolled studies was quantified using the Q-statistic and inconsistency index (I2). I2>50%, heterogeneity was considered as severe; if I2=25% to 50%, heterogeneity was considered as moderate, and if I^2^<25%, heterogeneity was considered as low. Pooled estimate of OR for risk of head and neck cancer was calculated using random effects model using the method of DerSimonian and Laird, with the estimate of heterogeneity being taken from the inverse-variance model. Forrest plot was made for pooled OR of included case control studies. Subgroup and sensitivity analysis were performed. All analyses were carried out in STATA software (Version 16.0; Stata Corporation, College Station, TX).

Publication Bias - Publication bias was analyzed and represented by a funnel plot, and funnel plot symmetry was assessed with Egger’s test (Figure 5). STATA software (Version 16.0; Stata Corporation, College Station, TX) was used for analysis.

## Results

Total 324 studies were identified through various databases (PubMed = 46, Google scholar = 36, Scopus = 242). After removal of duplicates, 262 studies were screened out of which 159 full text articles were assessed for eligibility and based on exclusion and inclusion criteria 145 studies were excluded. Out of the 145 studies which were excluded, in 101 studies risk of opium with cancer was not studied and in remaining 44 studies, risk of opium was studied with cancer other than head and neck cancer. 14 studies were included in data synthesis (11 case control studies and 3 cohort studies). 

Details of the studies included are given in Table 1. Studies are explained under the following heading: Author: year, country; Setting: Hospital/ Community, duration, study design; Participant Characteristics: cases, control, age, gender; Exposure Group Characteristics: Opium type, dose, frequency; Control Group Characteristics: Definitions; Outcome Characteristics: Type of Cancer, OR with 95% CI. Two studies Sheikh et al., (2019) and Khademi et al., (2012), were community-based studies while others were hospital based studies. Nasrollahzadeh et al., (2008), Tahami et al., (2014), Islami et al., (2004), and Khademi et al., (2013), described opium types into 5 categories includes teriak (crude opium), shireh (a refined opium extract), sukhteh (opium dross left in pipes after smoking opium), and heroin. Teriak and shireh can be smoked or ingested, sukhteh is usually ingested, and heroin is usually injected. Tahami et al., (2014), and Khademi et al., (2012) measured daily consumption of opium by a measurement unit, “Nokhod” (the local unit for opium use, equivalent to about 0.2 g)

Shakeri et al.,(2012), Nasrollahzadeh et al., (2008), Islami et al., (2004), and Pournaghi et al., (2019) studied the relationship between opium consumption and esophageal squamous cell carcinoma with odds ratio (OR) ranging from 1.37 to 2.1, whereas for oral cancer OR ranged from 4.09 to 5.01 as described in studies done by Fahmi et al., (1983), Razmpa et al.,(2014), and Saedi et al.,(2012). The risk of development of laryngeal cancer with opium consumption was highest and the OR ranged from 9.09 to 31.55 according to the studies done by Mausavi et al.,(2010), Berjis et al., (2017), and Bakhshae et al., (2017). There were three cohort studies namely by Aghcheli et al., (2011), Sheikh et al., (2019), and Khademi et al.,(2012) which reported hazard ratio ranging from 1.23 to 1.98 among opium users for the risk of esophageal squamous cell carcinoma.

**Figure 2:**
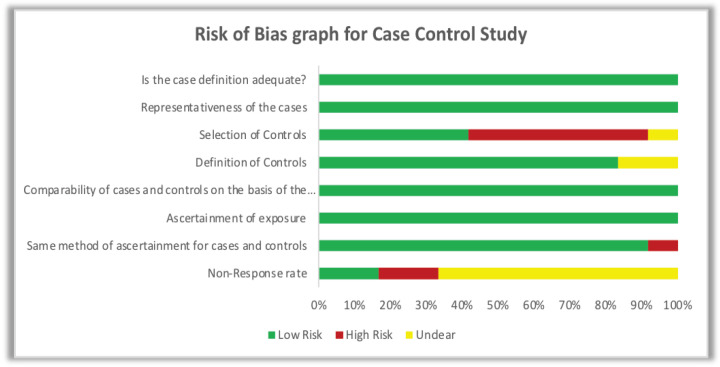
Risk of Bias Graph for Case Control Studies

Quality assessment of studies was done using New Castle Ottawa Scale. Details of risk of bias assessment is shown in detail in Figure 1, and Figure 2 for case control, and cohort studies respectively.

11 case control studies were included in synthesizing the results for meta-analysis. The pooled odds ratio for risk of cancer among opium users for the 11-case control study was 3.85 (2.18-6.79). Heterogeneity in the pooled estimate was high (I-squared=92.0%, Tau-squared=0.88). (Figure 4) In three cohort studies, hazard ratio for the risk of esophageal squamous cell carcinoma ranged from 1.23 to 1.98.

Publication bias: Figure 5, is showing the funnel plot. Publication bias was not present in this study. Both the Egger’s test and the Begg’s test for funnel plot asymmetry did not reach statistical significance (Egger test for small study effect showed non-significant result, p-value=0.104, Begg’s test, p-value =0.064).

*Sub-group analysis*

The high heterogeneity in the pooled estimate was further investigated through subgroup analysis. Highest OR was observed for the pooled estimate for risk of laryngeal cancer (19.98 (11.04-36.15)). Odds ratio for risk for esophageal cancer was observed as 1.66 (1.37-2.02), OR for risk of oral cavity cancer was observed as 3.06 (1.29-7.29). 

*Sensitivity analysis*

We have included only head and neck cancer for our systematic review and meta-analysis, but the study by Tahami et al., (2014) and Islami et al., (2004) included other upper GI cancers as well, therefore we performed sensitivity analysis dropping the study by Tahami et al., (2014) and Islami et al., (2004) which did not cause significant change in the pooled estimate. (pooled OR after removal of studies: 4.07 (2.08-7.94); compared to before 3.85 (2.18-6.79)). 

We also did sensitivity analysis by dropping small studies (sample size less than 200); however, the pooled estimate did not change after that also. (pooled OR after removal of studies: 3.66(2.01-6.67); compared to before 3.85 (2.18-6.79)).

## Discussion

Opioids, which include opiates (heroin and opium) and pharmaceutical and other synthetic opioids, are a major concern in many countries because of the severe health consequences associated with their use. In 2017, the use of opioids accounted for nearly 80 per cent of the 42 million years of “healthy” life lost as a result of disability and premature death (disability-adjusted life years, or DALYs) and 66 per cent of the estimated 167,000 deaths attributed to drug use disorders. 

Asia, host to more than 90 per cent of global illicit opium production and the world’s largest consumption market for opiates, accounted for almost 80 per cent of all opiates seized worldwide Middle East and South-West Asia and South Asia are two subregions that together account for almost 60 per cent of the estimated number of opiate users worldwide. The largest quantities of both opium and morphine seized were reported by the Islamic Republic of Iran, followed by Afghanistan and Pakistan. Iran is one of the topmost countries where largest quantities of opiates were seized.

A major drug use survey carried out recently in India found that in 2018, 2.1 percent of the population aged 10–75, a total of 23 million people, had used opioids in the past year. Among opioids, heroin is the most prevalent substance, with a past this is followed by the non-medical use of pharmaceutical opioids, and by opium at almost 0.5 per cent. Of the 23 million past-year opioid users, roughly one third, or 7.7 million people, suffer from opioid use disorders. Estimated levels of drug use disorders were: 0.1 per cent for opium use; 0.57 per cent for heroin use; and 0.23 per cent for use of pharmaceutical opioids. Compared with earlier estimates from a survey carried out in 2004, overall opioid use in India is estimated to have increased fivefold. 

Various mechanism has been proposed for the link between opium and different cancers (Ghahremani et al., 2019). The first potential mechanism is the genotoxic or mutagenic effect of opium smoke and pyrolysates, and some opium Alkaloids. During opium pyrolysis, multiple carcinogenic compounds are produced including heterocyclic and polycyclic aromatic hydrocarbons, primary aromatic amines, and N-nitrosamines, which can enter the body through the respiratory and digestive tracts and affect different organs (Malaveille et al., 1982).

Pyrolyzed derivatives and other mutagens containing hydroxy-phenanthrene moiety have been found in opium which have genotoxic and carcinogenic properties. (Friesen et al., 1985; Li and Lin, 1998). Sister chromatid exchange, and frame shift mutation have been observed in human peripheral blood lymphocytes and Salmonella typhimurium due to pyrolysis derived nitrogen containing heterocyclic components opium (Hewer et al., 1978; Malaveille et al., 1982; Perry et al., 1983). Morphine has also been found to be linked with carcinogenesis, by increasing methylation of DNA (Aliasgari et al., 2004; Friesen et al., 1985; Hewer et al., 1978; Hosseini et al., 2010; Karbakhsh et al., 2013; Li and Lin, 1998; Malaveille et al., 1982; Perry et al., 1983; Ribeiro Pinto and Swann, 1997). 

The second potential mechanism is through the tumor promoting effects of opiates (Grandhi et al., 2017) Opium has also been found to be associated with other aspects of carcinogenesis, like angiogenesis, epithelial-mesenchymal transition, and proliferation in animal models and human tissues (Gupta et al., 2002; Lennon et al., 2014; Moossavi et al., 2018).

The third potential mechanism is through facilitation of the effects of other carcinogens on different tissues, either by modifying the pharmacokinetics of these carcinogens and increasing their bioavailability or by impairing the physiological function of some organs and thus prolonging their exposure to the potential carcinogens (Li and Lin, 1998).

Other mechanism hypothesized as for carcinogenesis due to opium use is adulteration of opium with heavy metals during its preparation (Salehi et al., 2009; Soltaninejad and Shadnia, 2018).

These studies showed causal association between opium and cancer. 

Previous observational studies have shown association between opium addiction and higher risk of various types of cancers, namely pancreas (Moossavi et al., 2018), oral cavity (Fahmy et al., 1983; Lyons and Yazdi, 1969), esophagus (Malekzadeh et al., 2013; Nasrollahzadeh et al., 2008), stomach (Sadjadi et al., 2014; Shakeri et al., 2013), lung (MacLennan et al., 1977; Masjedi et al., 2013), larynx (Mousavi et al., 2010), and bladder (Aliasgari et al., 2004; Hosseini et al., 2010; Karbakhsh et al., 2013). Most of the studies showed different estimates for opium-only consumption, opium plus cigarette use, and opium use regardless of smoking status. Different types of opium, their methods of ingestions, duration of opium use, and the dose of opium, is associated with various cancers. 

A systematic review done by Afshari et al., (2017). Iran for opium use and risk of bladder cancer depicted the OR of 3.85 (3.05-4.87). Present meta-analysis reported higher OR for risk associated with development of head and neck cancer in opium users compared to non-users.

In another study done by Sheikh et al., (2019) Opium users have a significantly higher risk of developing cancers in different organs of the respiratory, digestive, and urinary systems and the CNS and that regular use of opiates might increase the risk of a range of cancer types. Use of opium was associated with an increased risk of developing all cancers combined (HR 1.40, 95% C.I. 1.24-1.58), gastrointestinal cancers (1.31, 1.11-1.55), and respiratory cancers (2.28, 1.58-3.30) in a dose-dependent manner. For site-specific cancers, use of opium was associated with an increased risk of developing esophageal (1.38, 1.06-1.80), gastric (1.36, 1.03-1.79), lung (2.21, 1.44-3.39), bladder (2.86, 1.47-5.55), and laryngeal (2.53, 1.21-5.29) cancers in a dose-dependent manner. High-dose opium use was associated with pancreatic cancer (2.66, 1.23-5.74). Ingestion of opium (but not smoking opium) was associated with brain (2.15, 1.00-4.63) and liver (2.46, 1.23-4.95) cancers in a dose-dependent manner.

In present study the pooled estimates from the eleven case control studies included shows approximately four-fold increase in the odds of developing head and neck cancer with the use of opium. 

This is the first systematic review and meta-analysis in the world, conducted on risk of head and neck cancer due to opium usage. The review provided an opportunity to examine the influence of opium on the risk of head and neck cancer. There was no evidence publication bias, which further increased the confidence in our estimate. 

There were certain limitations with the study mentioned which are given below. The heterogeneity was high which could be due to different methodologies, and different criteria for the exposure variable followed among studies included in the meta-analysis. Matching the case-control groups as well as the confounders adjusted during the odds ratio estimations were not same among the studies. Effect of opium should be estimated independently from tobacco smoking, which is a proven risk factor for many cancers. The results reported by the case-control studies might be prone to biases. It is better to investigate the associations between opium use and cancer by conducting longitudinal studies. We included three longitudinal studies in our current meta-analysis and in one of the cohort studies, the hazard ratio for risk of esophageal cancer among opium users was 1.85. Also, all the studies included were conducted in Iran. In the current metanalysis, we investigated did not consider the effect of the opium consumption methods. It should be taken into consideration by the future studies. The results of this meta-analysis can provide important information for the general population to protect themselves against a proven risk factor for one of the highly virulent cancers. People should not only prevent and stop smoking habits, but also avoid other behavioral risk factors such as opium consumption. 

In conclusion, this study showed that there was almost four-fold rise in risk of head and neck cancer among opium users compared to non-users. Although, there was large heterogeneity in the pooled estimate which could be due to difference in measure of opium usage. Also, the between-study variance was high which can be due to different type of head and neck cancers in different studies. 

Finally, Considering the increasing trend of opium consumption among populations especially in countries such as Iran which is in a critical gateway of opium substances, similar studies need to be carried out in India which is the largest manufacturer of legal opium for the pharmaceutical industry.

**Box 1:** Research Question Using PECOS Format

Criteria	Description
P: population	Patients with diagnosed head and neck cancer.
E: exposures	Exposure of interest was opium.
C: comparison	With versus without exposure of interest.
O: outcome	The primary outcome for the present study was risk estimate for head and neck cancer.
S: types of studies	All designs (cross-sectional, case-control, cohort and interventional studies), only quantitative studies.
Exclusion:	Grey literature, qualitative studies, reviews, meta-analyses, ecological studies, case series, case reports, policy papers, and comments. Papers published in language other than English. Non-peer reviewed articles. Studies published after 1st November 2019.

**Table 1:** Characteristics of Studies Included in This Review

S. No.	Author: year, country	Study setting, duration, study design	Participant characteristics: Cases, Controls, Age, Gender	Exposure Group Characteristics: Opium type, dose, frequency	Control Group Characteristics: Definitions	Outcome Characteristics: Type of Cancer, OR with 95% C.I.
1	Fahmi MS et al, 1982, Iran	Hospital based 16 years (1962-1978) Case control	Cases 381 [M: F 312: 69]; Controls 1000 [M:F 525: 475]	Not available	For 381 cases, 1000 persons of the same age group and socioeconomic status of the affected patients were taken	Oral Cancer, OR 5.01 (2.89-8.69)
2	Mausavi MRA et al, 2003, Iran	Hospital based 6 years (1996- 2002) Case control	Cases 98 [M:F 83:15]; Controls 312 [M:F 234:78]	Opium dependency was defined for one who was opium dependent for at least 5 years	98 patients with laryngeal cancer and 312 age and gender matched control subjects were selected	Laryngeal Cancer, OR 21.55 (10.54-44)
3	Islami F et al, 2004, Tehran, Iran	Hospital based 2 years (2001- 2003) Case control	Cases 238; Controls 260	Type - 1) shireh (opium sap), 2) sukhte (burned opium), Frequency – New user - started opium use during the year before the diagnosis was made. Old user - started opium use earlier than 1 year before the diagnosis	Out of total 238 patients, 260 patients were selected as control as people with no endoscopically suspicious lesion, (noncancer patients) which also had data for opium exposure	Esophageal (SCC+AC) + gastric cancer, OR 1.45 (0.99-2.11)
4	Nasrollahzadeh D et al, 2008, Iran	Hospital based 4 years (2003-2007) Case control	Cases 300 [M:F 150:150]; Controls 571 [M:F 278:293]	Type - 1) teriak (raw opium), 2) shireh (opium sap), 3) sukhte (burned opium), 4) heroin, Dose - opium at least once per week for a minimum of 6 months, Frequency - Categorized into three groups: no use, low use (<median use in controls), and high use (>median use).	For each case subject - two population based control of same residence or village, age (±2 years), and sex	Esophageal SCC OR, 1.95 (1.36-2.78)
5	Shakeri R et al 2012, Tehran, Iran	Hospital based 5 years (2002-2007) Case control	Cases 130 [M:F 79:51]; Controls 260 [M:F 158 102]	Type - not described, Dose - standardized opium consumption prevalence’s was 0.23 for the hospital controls	Two age- and sex-matched controls-hospitals	Esophageal SCC, OR 1.37 (0.85-2.21)
6	Saedi B et al 2012, Tehran, Iran	Hospital based 10 years (1999-2009) Case control	Cases 557 [M:F 219:338]; Controls 300 [M:F 189:111]	Opium dependency was defined for one who was opium dependent for at least 5 years	300 normal age- and sex-matched subjects with the same socioeconomic status were selected from normal individual	Oral Cancer, OR 4.31
7	Tahami AN et al 2014, Kerman, Iran	Hospital based 2 years (2010-2012) Case control	Cases 142 [M:F 104:38]; Controls 284 [M:F 208:76]	Types - 1) teriak (raw opium), 2) shireh (opium sap) Dose - Daily consumption of opium was measured by a measurement unit, “Nokhod” (Nokhod contains 0.2 grams). Frequency - Categorised into three groups: no use, low use (<median use in controls), and high use (>median use)	For each case subject - two control subjects matched to the case was selected	All UGI Cancer, OR 14.0 (4.7-47.5)
8	Razmpa E et al, 2014, Iran	Hospital based, 2 years (2008-2010) Case control	Cases 80 [M:F 51:29]; Controls 80 [M:F 50:30]	Opium dependency was defined for one who was opium dependent for at least 5 years	Eighty patients and 80 age and gender matched controls were selected.	Oral Cancer, OR 4.09 (1.2-13.6)
9	Berjis N et al, 2015, Iran	Hospital based, 1 years (2014-2015) Case control	Cases 180 [M:F 178:2]; Controls 180 [M:F 178:2]	Not available	180 patients and 180 age and gender matched controls were selected.	Laryngeal Cancer, OR 31.6 (7.9 – 43.6)
10A	Bakhshae M et al (A), 2010, Mashhad, Iran	Hospital based, 2 years (2008-2010) Case control	Cases 58; Controls 27	Opium use was defined as consuming the product at least once a day for a minimum of one year	Not available	Laryngeal Cancer, OR 9.09 (3.21-25.64)
10B	Bakhshae M et al (B), 2010, Mashhad, Iran	Hospital based, 2 years (2008-2010) Case control	Cases 95; Controls 27	Opium use was defined as consuming the product at least once a day for a minimum of one year	Not available	Upper Esophageal Cancer, OR 1.44 (0.57 -3.62)
11	Pournaghi SJ et al, 2017, Iran	Hospital based, 2 years (2013-2015) Case control	Cases- 96 [M:F 42:54]; Controls- 187 [M:F 81:106]	Not available	96 patients with esophageal SCC from cancer registry in North Khorasan. 187 controls were chosen from two general hospitals in North Khorasan.	Esophageal SCC, OR 2.1 (1.2-3.5)
S. No.	Author: year, country	Study setting, duration, study design	Participant characteristics: Cases, Controls, Age, Gender	Exposure Group Characteristics: Opium type, dose, frequency	Control Group Characteristics: Definitions	Outcome Characteristics: Type of Cancer, OR with 95% C.I.
12	Agcheli K et al, 2012, Golestan, Iran	Hospital Setting, 5 years (2002-2007) Cohort study	Participants, 426, [M:F 211:215]	Opium users - who had ever used the respective substances at least once per week for a minimum duration of 6 months. Opium non users - who had started using opium within one year before diagnosis of their disease	Not available	Esophageal SCC, HR 1.23 (0.98-1.54)
13	Khademi H et al 2012, Tehran, Iran	Community setting, 4 years (2004-2008) Cohort study	Participants, 50045, [M:F 21,230:28808]	Type - 1) teriak (raw opium), 2) shireh (opium sap), 3) sukhte (burned opium), 4) heroin, Dose - typical amount of use in nokhod (the local unit for opium use, equivalent to about 0.2 g), Frequency - days a week if weekly or more. The median daily amount of opium used was 0.6 g (25th-75th centile 0.2-1.2g).	50,045 participants aged 40- 75 were recruited at baseline.	Esophageal SCC, HR 1.98 (1.80-2.17)
14	Sheikh M et al 2019, Golestan, Iran	Community setting, 4 years (2004-2008), Cohort study	Participants, 50038, [M:F 21,230:28,808]; 317 developed Esophageal SCC, [M:F 174:143]	Types - opium consumption through smoking and ingestion was analyzed separately, Dose - typical amount of use in nokhod (the local unit for opium, use, equivalent to about 0.2 g), Frequency – never smoked opium, and for smokers, the tertiles of the cumulative nokhod-years of smoked opium, opium ingestion – never ingested opium or the 2 quantiles of the cumulative nokhod - years of ingested opium.	50,045 individuals, 40 to 75 years old, from urban and rural areas across Northeast Iran.	Esophageal SCC, HR 1.85 (1.18-2.90)

M:F, Male Female ratio; SCC, Squamous cell carcinoma; UGI, Upper Gastro-intestinal; AC, Adenocarcinoma

**Figure 3.**
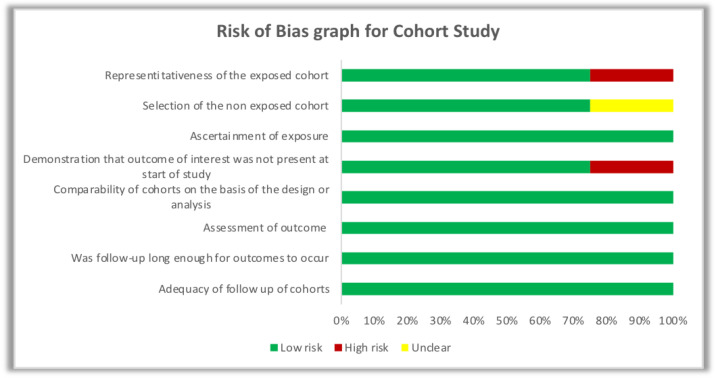
Risk of Bias Graph for Cohort Studies

**Figure 4:**
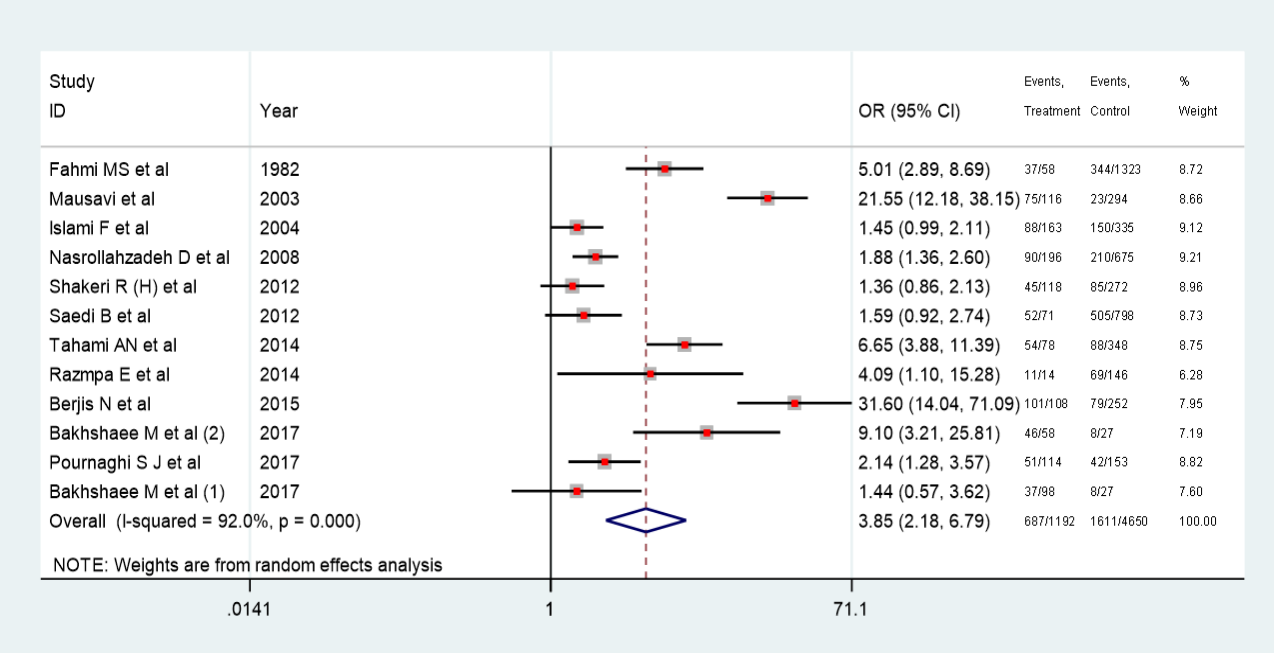
Point and Pooled Adjusted Odds Ratios for Head and Neck Cancer and Opium Consumption. In this plot, the size of each box indicated the weight of each primary study and the crossed lines indicating 95% confidence intervals. At the bottom of each plot, a diamond with a vertical dotted crossed line was designed indicating the pooled prevalence. The horizontal diameter of the diamond indicates the confidence interval of the pooled estimate. Another continuous vertical line in the plot crossed the number one shows the null hypothesis. The 95% confidence interval of each point odds ratio crossing this vertical line considered as non-significant effect

**Figure 5:**
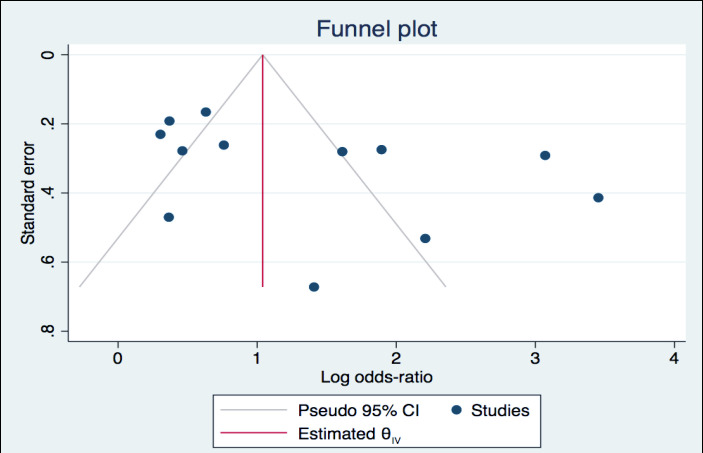
Funnel plot

**Figure 6:**
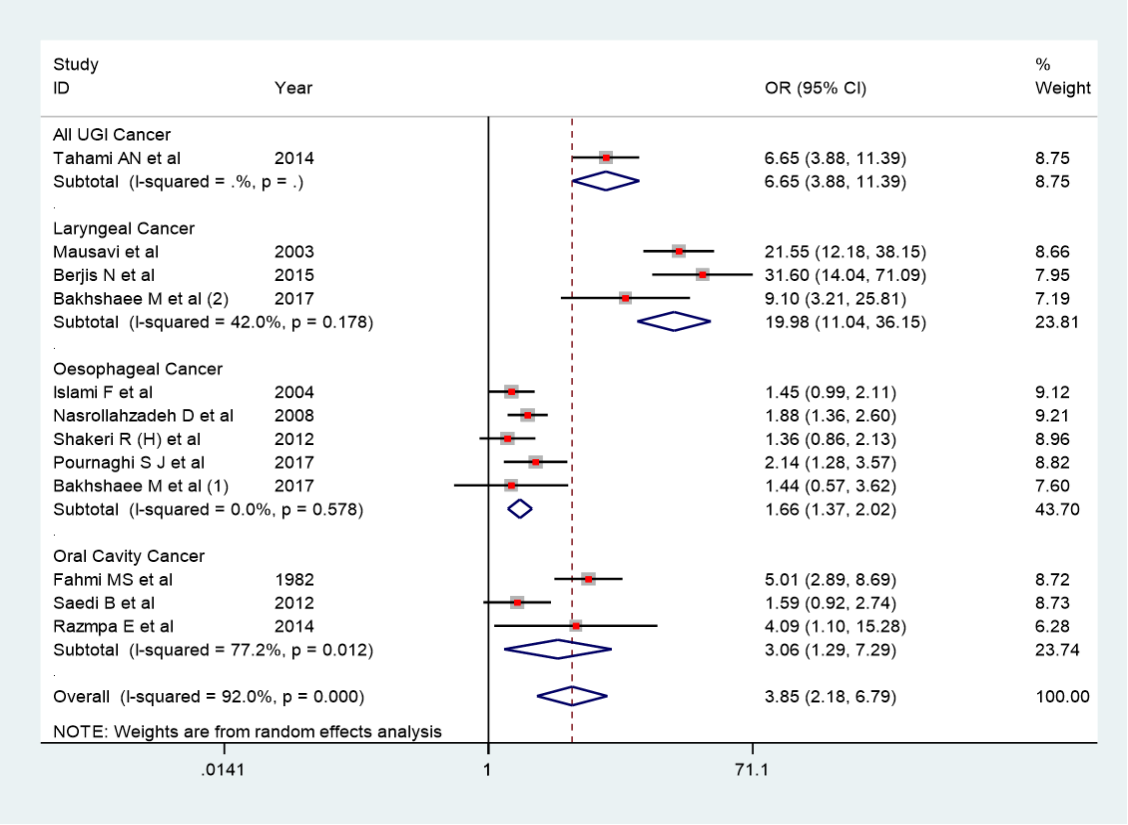
Subgroup Analysis According to Type of Cancer

## Author Contribution Statement

AJ and GS conceived and designed the study; AJ, GS, and ADG acquired data; AJ, GS, and ADG analyzed, and interpreted data; GS, and ADG drafted the initial manuscript. ADG, PRR, GS, and AJ performed critical revisions of the manuscript, and finalized the manuscript. All the authors approved the final version of the manuscript.

The protocol of this study was registered in PROSPERO (International prospective register of systematic reviews) at www.crd.york.ac.uk under the PROSPERO-ID CRD42020156049.
